# Biochemical evidence for Ku-independent backup pathways of NHEJ

**DOI:** 10.1093/nar/gkaa228

**Published:** 2020-04-02

**Authors:** Huichen Wang, Ange Ronel Perrault, Yoshihiko Takeda, Wei Qin, Hongyan Wang, George Iliakis

**Affiliations:** 1 Department of Radiation Oncology, Division of Experimental Radiation Oncology, Kimmel Cancer Center, Jefferson Medical College, Philadelphia, PA 19107, USA; 2 Institute of Medical Radiation Biology, Medical School of the University Duisburg-Essen, Essen, Germany; 3 Gene Regulation Program, Institute of Molecular Medicine and Genetics, Medical College of Georgia, Augusta, GA 30912, USA

*Nucleic Acids Research* (2003) 31(18): 5377–5388, doi:10.1093/nar/gkg728

The Authors and Editors wish to jointly publish an Expression of Concern regarding the above article.

The Editors wish to alert the readers that questions have been raised about the validity of some of the figures presented in this article.

In Figure 2B, some lanes have been duplicated as described below:

The sample of HeLa without NHS was lost, the authors duplicated the lane of HeLa with 0.05μl NHS.

The sample of M059-J with 0.2μl of NHS was lost, the authors duplicated the lane of M059-J with 0.025μl of NHS.

Lane 7 of Figure 3B (M059-J, probe only) has been copied from a different experiment.

While these irregularities may not fundamentally affect the results and conclusions of the article, the Editors nonetheless note that such manipulations of data and corresponding figures is unacceptable. The authors concur and deeply regret the transgression.

Keith Fox, Senior Executive Editor, Nucleic Acids Research

Barry Stoddard, Senior Executive Editor, Nucleic Acids Research

## Notes

Present address: Huichen Wang, Radiation Institute for Science and Engineering (RaISE), Prairie View A&M University, P.O. Box 519 MS2230, New Science building, Rm 322 Prairie View, TX 77446.

Present address: Ange Ronel Perrault, Yoh Scientific, Day and Zimmermann Company, Philadelphia, PA.

Present address: Yoshihiko Takeda, Ginza Hospital, Kabukiza Tower 16F, 4-12-15 Ginza, Chuo-ku, Tokyo, 104-0061 JAPAN.

Present address: Wei Qin, University of Pennsylvania, 12^th^ FL Endocrinology 3400 Civic CTR Blvd Bldg. Philadelphia, PA 19104.

Present address: Hongyan Wang, Sarah Bluff Lane, Cypress TX 77433 (home).

